# Supplementary material for: IMPOSE (IMProving Outcomes after Sepsis)—the effect of a multidisciplinary follow-up service on health-related quality of life in patients postsepsis syndromes—a double-blinded randomised controlled trial: protocol
Source: BMJ Open. 2014 May 24;4(5):e004966. doi: 10.1136/bmjopen-2014-004966 (PMC4039866; doi:10.1136/bmjopen-2014-004966)
Supplement: Author's manuscript [file bmjopen-2014-004966.draft_revisions.pdf]

IMPOSE: IMProving Outcomes after SEpsis: The effect of a multidisciplinary follow up service on health related quality of life in patients post sepsis syndromes, a double blinded randomized controlled trial: protocol

Journal:	*BMJ Open*
Manuscript ID:	bmjopen-2014-004966
Article Type:	Protocol
Date Submitted by the Author:	31-Jan-2014
Complete List of Authors:	Paratz, Jennifer; The University of Queensland, School of Medicine, Burns, Trauma & Critical Care Research centre; Royal Brisbane & Women's Hospital, Department of Intensive Care Medicine<br>Kenardy, Justin; University of Queensland, Psychology<br>Mitchell, Geoffrey; University of Queensland, School of Medicine<br>Comans, Tracy; Griffith University, School of Medicine<br>Coyer, Fiona; Queensland Institute of Technology, Nursing Studies<br>Thomas, Peter; Royal Brisbane & Womens Hospital, Physiotherapy<br>Boots, Robert; Royal Brisbane & Womens Hospital, Intensive Care Medicine
**Primary Subject Heading**:	Intensive care
Secondary Subject Heading:	Intensive care, Rehabilitation medicine
Keywords:	Adult intensive & critical care < ANAESTHETICS, REHABILITATION MEDICINE, Telemedicine < BIOTECHNOLOGY & BIOINFORMATICS

SCHOLARONE™ Manuscripts

IMPOSE: IMProving Outcomes after SEpsis: The effect of a multidisciplinary follow up service on health related quality of life in patients post sepsis syndromes, a double blinded randomized controlled trial: protocol

Investigators: Jennifer D Paratz[1,2,5,8], Justin Kenardy[3], Tracy Comans[4], Geoffrey Mitchell[6], Fiona Coyer[7], Peter Thomas[1,2,8], Robert J Boots [1,2]

[1]Burn, Trauma and Critical Care Research Centre, The University of Queensland; Brisbane, Australia [2]Department of Intensive Care Medicine, Royal Brisbane & Women's Hospital Brisbane, Australia; [3]CONROD, The University of Queensland Brisbane, Australia; [4]School of Medicine, Griffith University, Brisbane, Australia; [5]School of Rehabilitation, Griffith University, Brisbane, Australia [6]School of Medicine (Ipswich Campus), The University of Queensland, Ipswich, Australia; [7] Nursing Faculty, Queensland University of technology Brisbane, Australia; [8]Department of Physiotherapy, Royal Brisbane & Women's Hospital; Brisbane, Australia

Corresponding author:

Dr Jennifer Paratz

Chair and Research Fellow Burn,
Trauma and Critical Care Research Centre
Department of Intensive Care Medicine
Level 3, Ned Hanlon Building
Royal Brisbane and Women's Hospital
Butterfield Street, Brisbane, QLD, 4029
Telephone : 61 7 3646 1980
Facsimile : 61 7 36463542
Email: j.paratz@uq.edu.au

Key words: sepsis, septic shock, critical care, intensive care, weakness, post traumatic stress syndrome

Word Count: 2,063

Trial registration number: ACTRN12613000528752

For peer review only

ABSTRACT

Introduction: Patients post sepsis syndromes have been found to have poor quality of life and a high rate of recurring illness or mortality in the first year. Follow-up clinics have been instituted but evidence is sparse and there has been no clinic specifically for survivors of sepsis. The aim of this trial is to investigate if targeted screening and appropriate intervention to these subjects can result in an improved quality of life (short form (36) health survey (SF36v2™), decreased mortality in the first 12 months, decreased readmission to hospital and/or decreased use of health resources.

Methods and Analysis: 204 patients post sepsis syndrome and ventilated for greater than 48 hours will be randomized to one of two groups. The intervention group will attend an outpatient clinic two-monthly for six months and receive screening and targeted intervention. The usual care group will remain under the care of their physician. To analyse the results a baseline comparison will be done between each group. Generalized estimating equations (GEE) will compare the SF-36 domain scores between groups and across time points. Mortality will be compared between groups using a Cox proportional hazards analysis proportional hazards (time until death) analysis. Time to first readmission will be compared between groups by a survival analysis. Health care costs will be compared between groups using a generalized linear model (GLM). Economic (health resource) evaluation will be a within trial incremental cost utility analysis with a societal perspective.

Ethics and Dissemination: Ethical approval has been granted from Royal Brisbane & Womens Hospital Human Research Ethics Committee (HREC) (HREC/13/QRBW/17), The University of Queensland HREC (2013000543) and the

For peer review only

Australian Government Department of Health (26/2013). The results of this study will be submitted for publication in peer reviewed intensive care journal and presented at national and international intensive care and/or rehabilitation conferences.

For peer review only

Article Summary

Article Focus

- Survivors of critical illness have been noted to have physical, psychological and social problems for up to five years post discharge.
- The subgroups of patients with sepsis syndromes have been noted to have worse outcome compared to general intensive care patients
- No studies have investigated follow up for patients with sepsis syndromes post discharge from intensive care

Key Messages

- This study will investigate if providing screening and intervention to patients post sepsis syndrome will result in an improved quality of life, less readmission to hospital, less use of health resources and decreased mortality.

Strengths and limitations of this Study

- This is the first study to concentrate solely on patients with sepsis syndrome post intensive care discharge.
- There is a strong multidisciplinary team to provide a multitude of screening tools and interventions.
- This is a single centre study so lacks external generalizibility

For peer review only

BACKGROUND

Greater numbers of patients are surviving intensive care (ICU) but a new syndrome of "post intensive care syndrome" has appeared with residual physical, cognitive and psychosocial problems lasting from 5 to 15 years.[1,2] The rate of recurring major illness and/or mortality is also increased in the year after ICU discharge, with mortality ranging from 26-63% at one year post discharge in long stay ICU patients (≥ 48 hours).[3] ICU survivors additionally often have a number of problems [1-4] including debility and fatigue from loss of muscle mass, ongoing nutritional problems, difficulty with dealing with altered appearance and function, chronic pain [5] amnesia, and delusional symptoms All of these factors have a major impact on the health and productivity of survivors and carers, return to work rates, as well as the impact on availability of ICU and hospital beds, surgical waiting lists, health costs and society.[6] There has been a "call to arms" to provide improved management regimes to intensive care survivors.[7] All of these problems are particularly present in survivors of sepsis.[8]

Sepsis is defined as a powerful inflammatory response to severe infection. [9] The annual total costs of sepsis syndromes in the US are $16.7 billion nationally [10] with the incidence projected to increase by 1.5% per annum as patients develop more co-morbidities and resistant organisms. In brief, sepsis is a major public health concern.[11]

However the statistics above do not include the cost of ongoing disability and loss of productivity post illness. Patients with sepsis syndromes have significantly worse outcomes with lower health related quality of life (HRQoL)[12-15], particularly in the physical domain,[16,17] chronic pain [5,18] and cognitive domain[19] compared to non septic

For peer review only

ICU survivors. While other critically ill/injured or disabled patients eg burns, strokes, head injuries have established follow up regimes, patients with sepsis are not referred to formal rehabilitation and are discharged home with extremely poor function.

Follow up intensive care outpatient clinics are a recent innovation particularly within the United Kingdom. [20-22] These clinics identify and provide intervention for problems post hospital discharge including weakness, poor balance, impaired swallowing and nutrition, ongoing emotional problems and/or post-traumatic stress disorder in critical illness survivors. The one completed clinical trial on this topic [23] did not find a significant improvement in HRQoL. Researchers admitted that one discipline was not ideal and "further work should focus on the roles of early physical rehabilitation, delirium, cognitive dysfunction and relatives" Additionally the programme was conducted in a generic group of ICU survivors including those with only an overnight stay, and there may be greater discriminatory power in selecting one diagnostic group with a documented poor outcome such as sepsis syndromes. Additionally some countries (USA, Australia, South Africa) have major problems with large land area and relatively sparse population in rural areas leading to lack of resources and problems with follow up post intensive care. [24]

For peer review only

AIMS

The primary aim of this research is to investigate whether targeted follow up and intervention will improve health related quality of life (HRQoL) in survivors of sepsis syndromes. Secondary aims will be to investigate whether this intervention decreases readmission rates to hospital, 12 month mortality and health resource use.

This project will be an important investigation of follow up and management of the ICU patient in a sub population (sepsis) documented to have poor outcomes.[15] The project will incorporate a subgroup (rural patients) via telemedicine [25] in a novel method to overcome the problem of the "tyranny of distance" in follow up.

Hypotheses

The hypotheses are that post hospital follow up will improve health related quality of life, decrease mortality, economic and health resource use and readmission to hospital in patients post admission to intensive care with sepsis syndrome.

METHODS/DESIGN

This is a prospective, double blinded, interventional, repeated measures randomised controlled trial with concealed allocation, blinded assessors and intention to treat analysis. The study has been designed with the Consolidated Standards for Reporting of Trials guidelines (Figure 1). [26] The trial will be completed in two outpatient clinics.

One clinic will be located at Royal Brisbane & Womens Hospital, Brisbane, Australia; the other will be located at Bundaberg base Hospital, Bundaberg, Queensland, Australia

Subjects

Participants will be recruited from patients being discharged from a quaternary university-affiliated intensive-care unit at Royal Brisbane and Women's Hospital (RBWH), Brisbane, Australia. Participants will be randomized to one of two groups (intervention or control) post ICU discharge and just prior to discharge from hospital.

Inclusion criteria

Patients included will be male or female, ≥18 years, with a documented episode(s) of sepsis[9] episode(s) of sepsis (≥2 criteria of a systemic inflammatory response plus proven or strongly suspected infection, severe sepsis defined as sepsis plus organ failure, septic shock defined as severe sepsis not responding to management) and required mechanical ventilation for longer than 48 hours.

Exclusion criteria

Patients with neurological injuries, spinal injuries, and burns will be excluded, as these patients have existing rehabilitation programs and community support groups. Patients with haematological conditions or requiring palliative care post ICU would also be excluded.

For peer review only

Randomisation

Participants will be randomised by opaque sealed envelope after enrolment. Numbers in the envelope will be generated by a computer-generated randomisation (http://www.randomization.com) table based on blocks of four to assign patients to intervention or usual care group. The randomization sequence will be concealed from consent designee staff and protected by an electronic password. Participants will be stratified based on a score > 3 on the Cumulative Illness rating Scale (CIRS-G) [27] We are stratifying patients as while most patients who develop sepsis syndromes have a number of co-morbidities, there can be a small number of relatively young, healthy patients who are affected.

INTERVENTION

Intervention Group

Patients in the intervention group will attend a follow-up clinic two-monthly for up to six months after discharge from the hospital. Screening instruments will be utilized on the first visit and appropriate management and referral provided. These will involve: overall medical review involving all systems assessment, medications, vital signs, muscle strength [28], assessment of mobility (*modified Rivermead Mobility Index,*[29] balance assessment (*Berg Balance measure)*[30], discussion with carer as to concerns (*Zarit Burden interview),* [31]screening for chronic pain syndromes (*Brief Pain inventory*), [32] nutritional review , screening with the *(PTSD) Checklist-Civilian Version* (for post traumatic stress syndrome) [33] , *generalized anxiety disorder (GAD-*

For peer review only

7) [34] and *The Patient Health Questionnaire-9 for depression* [35]. Following the results of the screening and team discussion subjects and/or carer will be referred to appropriate agencies including general practitioner, specialist, physiotherapist, nutritionist, speech pathologist, exercise physiologist, psychologist and/or occupational therapist. These instruments are screening for post intensive care problems that have been identified in previous studies.[12-19] Members of the research team include an intensivist/thoracic physician, two specialist respiratory physiotherapists, a psychologist, a senior nurse researcher, a professor of community and general practice and a health economist/community physiotherapist.

Standard Care

Patients in the control group will have usual care ie referred to specialist outpatient clinics and/or general practitioners on discharge.

Sub Group - rural patients

A small number of patients (10) originally managed in the major quaternary level intensive care unit will be discharged to a rural area. We will test the feasibility of providing follow-up care to these patients. The patients will also be randomized to either an intervention or usual care group. The patients w ill be interviewed via telemedicine (videoconferencing) [25] and a research assistant appointed to complete other screening tools and refer the patient to appropriate agencies. If specialist services are required eg chronic pain clinic, this may also be accessed via telemedicine.

For peer review only

OUTCOME MEASURES

Primary measure

The primary measure will be health related quality of life (HRQoL) as measured by the short from (36) health survey version 2 (SF36v2™)[36], which demonstrates content and construct validity, sensitivity and responsiveness to change in many patient groups[37] including intensive care survivors and post sepsis.[38] Pilot testing of the SF-36 in this population by this team has been conducted in a current trial of inpatients post sepsis and has shown excellent completion rates at six months by telephone.[39]

Secondary measures

Patients' readmission rates to hospital (medical record data), 12 month mortality (trend expected only) and economics and health care resource use. The latter will be monitored through examination of patient medical records, patient self-report and through Medicare / Pharmaceutical Benefits Scheme data extraction. The SF-6D[40] is a preference based six dimensional health state measure suitable for economic evaluation that is derived from the SF-36 The SF-6D defines 249 health states and a summary utility score for the SF-6D will be calculated using UK weights Individual patient productivity will be measured using a work/activity impairment questionnaire, the Work Productivity and Activity Impairment Questionnaire (WPAI).[41]

For peer review only

The outcome measure of SF36v2™ will be taken at baseline, six and twelve months post enrolment in the study. All other measures (mortality, hospital readmission and economic and health resource use) will be taken at 6 and 12 months post enrolment.

Sample size

Power calculations are based on SF-36 using previously published Minimally Clinically Important Differences (MCIDs) for the SF36 physical domain scores of 7.8 points (sd 15) in our pilot trial on rehabilitation in sepsis.[39] The sample size, based on a two sample comparison of means was calculated at 78 per group for the SF-36 at 90% power with a two-sided, alpha error level of 0.05. Allowing for an almost 30% loss to follow up (mortality, drop outs) a total of 204 participants will be required.[42]

Planned Statistical analysis

Primary outcome

A baseline comparison of demographics, severity of illness acute physiological and chronic health evaluation score (APACHE II), [43] and baseline measures will be done between each group, using a combination of t-tests and Chi-square.

Primary outcome: Generalized estimating equations (GEE) will be used to compare the SF-36 domain scores between groups and across time points. The GEE is a flexible technique that takes into account the correlations within individuals in longitudinal study designs and can account for missing data without need for imputation techniques [44]

Secondary outcomes

For peer review only

Mortality will be compared between groups using a Cox proportional hazards (time until death) analysis proportional hazards (time until death) analysis. [45] Time to first readmission will be compared between groups by a survival analysis. Utility estimates derived from the SF-6D will be analysed using a GEE. Health care costs will be compared between groups using a generalized linear model (GLM) to account for the non normal distribution. Economic (health resource) evaluation will be a within trial incremental cost utility analysis with a societal perspective.[46] Direct costs included will be: cost of care provided in hospitals, by general practitioners, home nursing, complementary [allied] health providers, alternative health providers, opportunity cost of unpaid carers, cost of transportation to program and to other health services, and cost of pharmaceuticals. Indirect costs include loss of productivity due to disease, such as time lost from work or providing informal care to another individual. All costs will be valued using market costs where available and productivity costs will be valued using actual patient wage rates.

The sub group of rural patients will be analysed both in the full analysis and as a sub group results compared to metropolitan patients.

Data management

A custom designed data-base will store de-identified patient data on a secure password protected file. This will be entered from hard copies of the scoring sheets. Data will be reviewed by research office staff twice yearly and cross referenced with stored hard copies.

For peer review only

Contingencies

If subject recruitment does not reach the required sample size by two years, the study can be extended for a further one to two years.

Methods for protecting against other sources of bias

This study will be double blinded with the assessors and a number of investigators blinded from the group allocation. There will be concealed allocation of subjects. The subjects will be stratified for Cumulative Illness rating Scale (CIRS-G). [27] Subjects when being consented will be told they will in one of two groups who will both be followed up post hospital and may need to attend an outpatient clinic. Patients are unaware of the usual follow up post hospital so should not feel they are receiving an increased/decreased level of care. Both groups will be contacted by telephone for completion of HRQoL life instruments at the required stages. Subjects may have varying severity of illness, lengths of stay and/or time on mechanical ventilation in intensive care, however this will be compared between groups and if unequal adjusted models using the relevant covariates will be presented as well as unadjusted comparisons.

A few subjects in either group may be referred to rehabilitation by their treating physician. This will be compared between groups. A number of patients may be uncontactable for final outcome measures however we have had 95 percent rate in a current trial at six months post sepsis. [39] The GEE [44] will allow for missing data. If patients "cross over" ie do not receive the planned intervention, they will still be analysed with the group they originally enrolled as per intention to treat.

For peer review only

ETHICS AND DISSEMINATION

The trial has been registered on the Australian and New Zealand Clinical Trials Registry (ACTRN12613000528752). Ethical approval has been granted from Royal Brisbane & Womens Hospital Human Research Ethics Committee (HREC) (HREC/13/QRBW/17), The University of Queensland HREC (2013000543) and the Australian Government Department of Health (26/2013).

Any adverse events connected to the trial would be immediately reported to one of the above three committees.

The results of this study will be submitted for publication in peer reviewed intensive care journal and presented at national and international intensive care and/or rehabilitation conferences.

CONCLUSION

Follow-up post intensive care stay is presently a topic of great interest with a realization that surviving critical illness may result in poor quality of life. Patients who have had severe sepsis syndromes have been shown to have a poorer quality of life and outcome than generic intensive care patients. This study will investigate whether targeted intervention from a multidisciplinary team will result in benefits and is economically feasible for this group of patients.

For peer review only

Competing interests: Nil

Funding: Funding has been provided for this study from the Royal Brisbane and Womens' Hospital Research Foundation Research foundation ($40,000) and the Physiotherapy Research Fellowship, Health and Medical Research, Queensland Government ($182,000).

Contributorship: All authors participated in the manuscript preparation, writing and editing.

For peer review only

References

1. Oeyen SG, Vandijck DM, Benoit DB et al. Quality of life after intensive care: a systematic review of the literature. *Crit Care Med* 2010;38:2386-400

2. Myhren H, Ekeberg O, Stokland O. Health related quality of life and return to work after critical illness in general intensive care unit patients: a 1-year follow up study. *Crit Care Med 2010*;38:1554-61

3. Williams TA, Dobb GJ, Finn JC et al. Long term survival from intensive care: a review. *Intensive Care Med* 2005;311306-15

4. Desai SV, Law TJ, Needham DM. Long-term complications of critical care. *Crit Care Med* 2011;39:371-9

5. Battle CE, Lovett S, Hutchings H. Chronic pain in survivors of critical illness: a retrospective analysis of incidence and risk factors *Crit Care* 2013, 17:R101 http:// ccforum.com.contnet/17/3/R101

6. Griffiths J Hatch RA, Bishop J et al. An exploration of social and economic outcome and health related quality of life after critical illness in general intensive care unit survivors: a 12-month follow-up study *Crit Care* 2013; 17:R100, http://ccforum.com/content/17/3/R100

7. Needham DM, Feldman DR, Kho ME. The functional costs of ICU survivorship. Collaborating to improve post-ICU disability. *Am J Respir Crit Care Med* 2011;183:1037-42

8. Hofhuis JGM, Spronk PE, van Stel HF et al. The impact of severe sepsis on health related quality of life: a long term follow up study. *Critical Care and Trauma* 2008;107:1957-64

9. Levy MM, Fink MP, Marshall JC et al. 2001 SCCM/ESICM/ACCP/ATS/SIS International Sepsis Definitions Conference. *Intensive Care Med* 2003;29:530–8

10. Angus DC, Lind-Zwirble WT, Lidicker J et al. Epidemiology of severe sepsis in the United States analysis of incidence outcome, and associated costs. *Crit Care Med* 2001;29:1303-10

For peer review only

11. Silva E, Araujo DV. Economic and Social Burden of Severe Sepsis. In Yearbook of Intensive Care and Emergency Medicine 2009:129-38

12. Karlsson S, Ruokonen E, Varpula T et al. Long-term outcome and quality adjusted life years after severe sepsis. *Crit Care Med* 2009; 37: 1268-74

13. Zhang K, Mao X, Fang Q, et al. Impaired long-term quality of life in survivors of severe sepsis Chinese multicenter study over 6 years. *Anaesthesist* 2013; 62:995–1002

14. Cuthbertson BH, Elder A, Hall S, et al. Mortality and quality of life in the five years after severe sepsis. Critical Care 2013, 17:R70 http://ccforum.com/content/17/2/R70

15. Winters BD, Eberlein M, Leung J et al. Long-term mortality and quality of life in sepsis: a systematic review .*Crit Care Med* 2010;38:1276-83

16. Paratz JD, Kayambu G. Early exercise and attenuation of myopathy in the patient with sepsis in ICU. *Phys Ther Rev* 2011;16:58-65

17. Baldwin CE, Bersten AD. Alterations in respiratory and limb muscle strength and size in patients with sepsis who are mechanically ventilated. *Phys Ther* 2014;94:68-82.

18. Zimmer A, Rothaug J, Mescha S, et al. Chronic pain after surviving sepsis. *Crit Care* 2006: 10(suppl 1):421.

19. Iwashyna TJ, Ely EW, Smith DM et al. Long-term cognitive impairment and functional disability among survivors of severe sepsis. *JAMA* 2010;304: 1787-94

20. Griffiths JA, Barber VS, Cuthbertson BH et al. A national survey of intensive care follow-up clinics. *Anaesthesia* 2006;61:950–5

21. Bambi S, Solaro M, Cianchi G et al. Outpatient Intensive Care Unit Follow-Up Services in Italy: A National Survey A Brief Report. *Dimens Crit Care Nurs* 2013;32:46-9

22. Williams TA, Leslie GD. Beyond the walls: A review of ICU clinics and their impact on patient outcomes after leaving hospital. *Aust Critical Care* 2008;21:6-17

23. Cuthbertson BH, Rattray J, Campbell MK et al. The PRaCTICal study of nurse led, intensive care follow up programmes for improving long term outcomes from critical illness: a pragmatic randomised controlled trial. *BMJ* 2009; 339:b3723. doi: 10.1136/bmj.b3723.

24. Williams TA, Leslie GD. Challenges and possible solutions for long-term follow-up of patients surviving critical illness. *Aust Crit Care* 2011;24:175-85

For peer review only

25. Boots RJ, Singh S, Terblanche M et al. Remote care by telemedicine in the ICU: many models of care can be effective. *Curr Opin Crit Care*. 2011;17:634-40

26. Moher D, Schulz K, Altman D. The CONSORT statement: revised recommendations for improving the quality of parallel–group randomized controlled trials. *Ann Internal Med* 2001;134:657-62

27. Waldman E, Potter JF. A prospective evaluation of the Cumulative Illness Rating Scale. *Aging* 1992;4:171-8.

28. Baldwin CE, Paratz JD, Bersten AD. Muscle strength assessment in critically ill patients with handheld dynamometry: An investigation of reliability, minimal detectable change, and time to peak force generation. *J Crit Care* 2012;28:77-86

29. Lennon S, Johnson L. The modified Rivermead Mobility Index: validity and reliability. *Disabil Rehabil*. 2000;22:833–9.

30. Thorbahn LD, Newton RA. Use of the Berg Balance test to predict falls in elderly persons. *PTJ*. 1996;76:576–85.

31. Zarit SH, Reever KE, Bach-Peterson J. Relatives of the impaired elderly: correlates of feelings of burden. *Gerontologist* 1980;20:649-55.

32. Cleeland CS. Measurement of pain by subjective report. In: Chapman CR, Loeser JD, editors. *Advances in Pain Research and Therapy*, Volume 12: Issues in Pain Measurement. New York: Raven Press; 1989:391-403.

33. Conybeare D, Behar E, Solomon A et al. The PTSD Checklist—Civilian Version: Reliability, Validity, and Factor Structure in a Nonclinical Sample. *J Clin Psychol* 2012;68:699-713

34. Spitzer RL, Kroenke K, Williams JBW et al. A brief measure for assessing generalized anxiety disorder. *Arch Intern Med*. 2006;166:1092-7.

35. Kroenke K, Spitzer RL, Williams JBW. The PHQ-9 Validity of a Brief Depression Severity Measure. *J Gen Intern Med* 2001;16:606–13.

36. Ware JD, Sherbourne CD. The MOS 36-item short-form health survey (SF-36). I. Conceptual framework and item selection. *Medical Care* 1992;30:473-83.

37. Contopoulos-Ioannidis DG, Karvouni A, Kouri I et al. Reporting and interpretation of SF-36 outcomes in randomised trials: systematic review. *BMJ,* 2009; 338: doi: 10.1136/bmj.a3006.a3006.

38. Heyland DK, Hopman W, Coo H et al. Long-term health-related quality of life in survivors of sepsis. Short Form 36: A valid and reliable measure of health-related quality of life. *Crit Care Med* 2000;28:3599-605.

For peer review only

39. Kayambu G, Boots R, Paratz J. A Prospective Randomised Controlled Trial investigating Functional and Physiological Outcomes following Early Rehabilitation in Sepsis: The i-PERFORM Trial - Protocol Article. *BMC Anesthesiology* 2011;11:21. doi: 10.1186/1471-2253-11-21

40. Brazier J, Roberts J, Deverill M. The estimation of a preference-based measure of health from the SF-36. *J Health Economics* 2002;21:271-92.

41. Reilly MC, Zbrozek AS, Dukes E. The validity and reproducibility of a work productivity and activity impairment measure. *PharmacoEconomics* 1993; 4:353-65.

42. Cohen J. Statistical power analysis for the behavioral sciences. Hillsdale, NJ, Erlbaum 1988.

43. Knaus WA, Draper EA, Wagner DP et al. APACHE II: a severity of disease classification system. *Crit Care Med* 1985;13: 818–29

44. Twisk, J. de Vente W. Attrition in longitudinal studies. How to deal with missing data. *J Clin Epidemiol 2002;* 55:329-37

45. Schoenfeld D. Sample-size formula for the proportional-hazards regression model. *Biometrics* 1983;39:499-503.

46. Drummond M, O'Brien B, Stoddart G et al. methods for the economic evaluation of health care programmes. Oxford medical Publications. New York, 1993

For peer review only

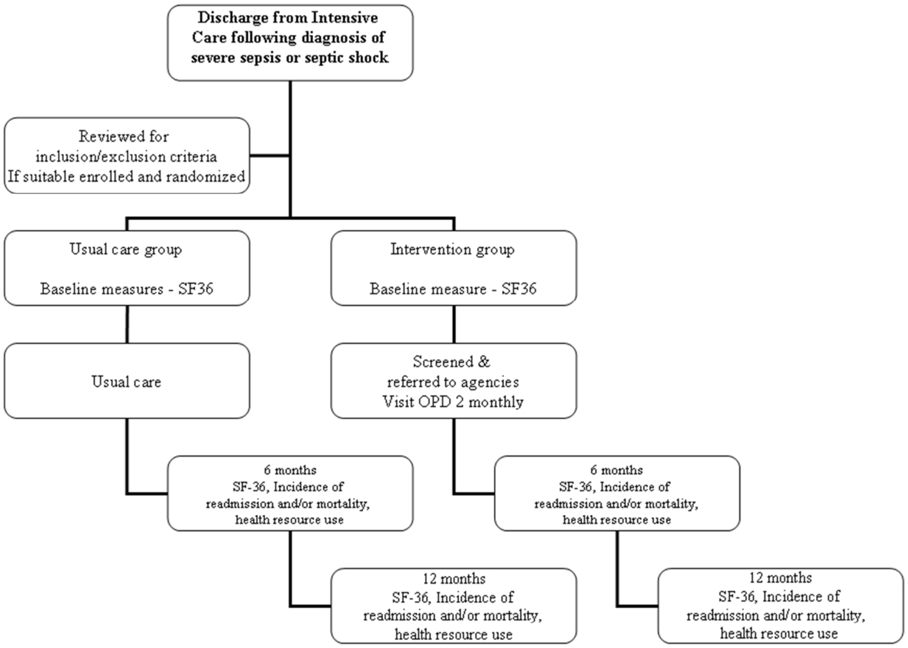

Figure 1 Consort Flow Diagram
254x190mm (96 x 96 DPI)

For peer review only

IMPOSE: IMProving Outcomes after SEpsis: The effect of a multidisciplinary follow up service on health related quality of life in patients post sepsis syndromes, a double blinded randomized controlled trial: protocol

Journal:	*BMJ Open*
Manuscript ID:	bmjopen-2014-004966.R1
Article Type:	Protocol
Date Submitted by the Author:	29-Apr-2014

Complete List of Authors:	Paratz, Jennifer; The University of Queensland, School of Medicine, Burns, Trauma & Critical Care Research centre<br>Kenardy, Justin; University of Queensland, Psychology<br>Mitchell, Geoffrey; University of Queensland, School of Medicine<br>Comans, Tracy; Griffith University, School of Medicine<br>Coyer, Fiona; Queensland Institute of Technology, Nursing Studies<br>Thomas, Peter; Royal Brisbane & Womens Hospital, Physiotherapy<br>Singh, Sunil; Bundaberg Base Hospital, Intensive Care Unit<br>Luparia, Louise; University of Queensland, School of Medicine<br>Boots, Robert; Royal Brisbane & Womens Hospital, Intensive Care Medicine

**Primary Subject Heading**:	Intensive care
Secondary Subject Heading:	Intensive care, Rehabilitation medicine
Keywords:	Adult intensive & critical care < ANAESTHETICS, REHABILITATION MEDICINE, Telemedicine < BIOTECHNOLOGY & BIOINFORMATICS

SCHOLARONE™ Manuscripts

For peer review only

IMPOSE: IMProving Outcomes after SEpsis: The effect of a multidisciplinary follow up service on health related quality of life in patients post sepsis syndromes, a double blinded randomized controlled trial: protocol

Investigators: Jennifer D Paratz[1,2,5,8], Justin Kenardy[3], Geoffrey Mitchell[6], Tracy Comans[4], Fiona Coyer[7], Peter Thomas[1,2,8], Sunil Singh[9], Louise Luparia[1,5], Robert J Boots [1,2]

[1]Burn, Trauma and Critical Care Research Centre, The University of Queensland; Brisbane, Australia [2]Department of Intensive Care Medicine, Royal Brisbane & Women's Hospital Brisbane, Australia; [3]CONROD, The University of Queensland Brisbane, Australia; [4]School of Medicine, Griffith University, Brisbane, Australia; [5]School of Rehabilitation Sciences, Griffith University, Brisbane, Australia [6]School of Medicine (Ipswich Campus), The University of Queensland, Ipswich, Australia; [7] Nursing Faculty, Queensland University of Technology Brisbane, Australia; [8]Department of Physiotherapy, Royal Brisbane & Women's Hospital; Brisbane, Australia, [9]Intensive Care Unit, Bundaberg Hospital, Bundaberg, Australia

Corresponding author:

Dr Jennifer Paratz

Chair and Senior Research Fellow
Burn, Trauma and Critical Care Research Centre
Department of Intensive Care Medicine
Level 3, Ned Hanlon Building
Royal Brisbane and Women's Hospital
Butterfield Street, Brisbane, QLD, 4029
Telephone: 61 7 3646 1980
Facsimile: 61 7 36463542
Email: j.paratz@uq.edu.au

Key words: sepsis, septic shock, critical care, intensive care, weakness, post traumatic stress syndrome

Word Count: 2,507

Trial registration number: ACTRN12613000528752

For peer review only - http://bmjopen.bmj.com/site/about/guidelines.xhtml

ABSTRACT

Introduction: Patients post sepsis syndromes have poor quality of life and a high rate of recurring illness or mortality. Follow-up clinics have been instituted for patients post general intensive care but evidence is sparse and there has been no clinic specifically for survivors of sepsis. The aim of this trial is to investigate if targeted screening and appropriate intervention to these subjects can result in an improved quality of life (Short Form -36 health survey (SF36v2), decreased mortality in the first 12 months, decreased readmission to hospital and/or decreased use of health resources.

Methods and Analysis: 204 patients post sepsis syndrome will be randomized to one of two groups. The intervention group will attend an outpatient clinic two-monthly for six months and receive screening and targeted intervention. The usual care group will remain under the care of their physician. To analyse the results a baseline comparison will be done between each group. Generalized estimating equations (GEE) will compare the SF-36 domain scores between groups and across time points. Mortality will be compared between groups using a Cox proportional hazards analysis proportional hazards (time until death) analysis. Time to first readmission will be compared between groups by a survival analysis. Health care costs will be compared between groups using a generalized linear model (GLM). Economic (health resource) evaluation will be a within trial incremental cost utility analysis with a societal perspective.

For peer review only - http://bmjopen.bmj.com/site/about/guidelines.xhtml

Ethics and Dissemination: Ethical approval has been granted from Royal Brisbane & Womens Hospital Human Research Ethics Committee (HREC) (HREC/13/QRBW/17), The University of Queensland HREC (2013000543), Griffith University (RHS/08/14/HREC) and the Australian Government Department of Health (26/2013). The results of this study will be submitted to peer reviewed intensive care journals and presented at national and international intensive care and/or rehabilitation conferences.

Australian and New Zealand Clinical Trials Registry ACTRN12613000528752

For peer review only

For peer review only - http://bmjopen.bmj.com/site/about/guidelines.xhtml

Article Summary

Article Focus

- Survivors of critical illness have been noted to have physical, psychological and social problems for up to five years post discharge.
- The subgroup of patients with sepsis syndromes have been noted to have worse outcome compared to general intensive care patients
- No studies have investigated targeted intervention for patients with sepsis syndromes post discharge from intensive care

Key Messages

- This study will investigate if providing screening and intervention to patients post sepsis syndrome will result in an improved quality of life, less readmission to hospital, less use of health resources and decreased mortality.

Strengths and limitations of this Study

- This is the first study to concentrate solely on patients with sepsis syndrome post intensive care discharge.
- There is a strong multidisciplinary team to provide a multitude of screening tools and interventions.
- Individualized management will be provided to patients.
- This is a single centre study so lacks external generalizibility

For peer review only

BACKGROUND

Greater numbers of patients are surviving intensive care (ICU) but a new syndrome of "post intensive care syndrome" has appeared with residual physical, cognitive and psychosocial problems lasting from 5 to 15 years.[1,2] The rate of recurring major illness and/or mortality is also increased in the year after ICU discharge, with mortality ranging from 26-63% at one year post discharge in long stay ICU patients (≥ 48 hours).[3] ICU survivors additionally often have a number of problems [1-4] including debility and fatigue from loss of muscle mass, ongoing nutritional problems, difficulty with dealing with altered appearance and function, chronic pain [5] amnesia, and delusional symptoms All of these factors have a major impact on the health and productivity of survivors and carers, return to work rates, as well as the impact on availability of ICU and hospital beds, surgical waiting lists, health costs and society.[6] There has been a "call to arms" to provide improved management regimes to intensive care survivors.[7] The NICE clinical guidelines [8] provided by the NHS National Institute of Health and Clinical Excellence recommend "optimisation of recovery" rather than merely survival post intensive care. All of these problems are particularly present in survivors of sepsis.[9]

Sepsis is defined as a powerful inflammatory response to severe infection. [10] The annual total costs of sepsis syndromes in the US are $16.7 billion nationally [11] with the incidence projected to increase by 1.5% per annum as patients develop more co-morbidities and resistant organisms. In brief, sepsis is a major public health concern.[12]

For peer review only

For peer review only - http://bmjopen.bmj.com/site/about/guidelines.xhtml

However the statistics above do not include the cost of ongoing disability and loss of productivity post illness. Patients with sepsis syndromes have significantly worse outcomes with lower health related quality of life (HRQoL)[13-16], particularly in the physical domain,[17,18] chronic pain [5,19] and cognitive domain [20] compared to non-septic ICU survivors. While other critically ill/injured or disabled patients eg burns, strokes, head injuries have established follow up regimes, patients with sepsis are not referred to formal rehabilitation and are discharged home with extremely poor function.

Follow up intensive care outpatient clinics are a recent innovation particularly within the United Kingdom. [21-23] These clinics identify and provide intervention for problems post hospital discharge including weakness, poor balance, impaired swallowing and nutrition, ongoing emotional problems and/or post-traumatic stress disorder in critical illness survivors. The one completed clinical trial on this topic [24] did not find a significant improvement in HRQoL. Researchers admitted that one discipline was not ideal and "further work should focus on the roles of early physical rehabilitation, delirium, cognitive dysfunction and relatives". The programme was conducted in a generic group of ICU survivors including those with only an overnight stay, and there may be greater discriminatory power in selecting one diagnostic group with a documented poor outcome such as sepsis syndromes. Additionally some countries (Canada, Australia, South Africa) have major problems with large land area and relatively sparse population in rural areas leading to lack of resources and problems with follow up post intensive care. [25]

AIMS

For peer review only

The primary aim of this research is to investigate whether targeted follow up and intervention will improve health related quality of life (HRQoL) in survivors of sepsis syndromes. Secondary aims will be to investigate whether this intervention decreases readmission rates to hospital, 12 month mortality and health resource use.

This project will be an important investigation of follow up and management of the ICU patient in a sub population (sepsis) documented to have poor outcomes.[16] The project will incorporate a subgroup (rural patients) via telemedicine [26] in a novel method to overcome the problem of the "tyranny of distance" in follow up.

Hypotheses

The hypotheses are that post hospital follow up will improve health related quality of life, decrease mortality, economic and health resource use and readmission to hospital in patients post admission to intensive care with sepsis syndrome.

METHODS/DESIGN

This is a prospective, double blinded, interventional, repeated measures, superiority, randomised controlled trial with concealed allocation, blinded assessors and intention to treat analysis. The study has been designed with the Consolidated Standards for Reporting of Trials guidelines [27] (Figure 1) and the Standard Protocol items: Recommendation for Interventional Trials (SPIRIT 2013). [28] The trial will be completed in two outpatient clinics. One clinic will be in the outpatient department located at a university-affiliated tertiary hospital Royal Brisbane & Womens Hospital,

For peer review only

Brisbane, Australia; the other will be located at a regional hospital, the Bundaberg Base Hospital, Bundaberg, Queensland, Australia

Subjects

Participants will be recruited from patients being discharged from a quaternary university-affiliated intensive-care unit at Royal Brisbane and Women's Hospital (RBWH), Brisbane, Australia. Participants will be randomized to one of two groups (intervention or control) post ICU discharge and just prior to discharge from hospital.

Inclusion criteria

Patients included will be male or female, ≥18 years, with a documented episode(s) of sepsis [10] episode(s) of sepsis (≥2 criteria of a systemic inflammatory response plus proven or strongly suspected infection, severe sepsis defined as sepsis plus organ failure, septic shock defined as severe sepsis not responding to management) and required respiratory support for longer than 48 hours. Enrolment in the study should be within one month of discharge from hospital.

Exclusion criteria

Patients with neurological injuries, spinal injuries, and burns will be excluded, as these patients have existing rehabilitation programs and community support groups. Patients with haematological conditions or requiring palliative care post ICU would also be excluded. Those or mental disabilities that patients with psychiatric

For peer review only - http://bmjopen.bmj.com/site/about/guidelines.xhtml

and/mental disability that preclude them understanding the questionnaires and non-English speaking patients would be excluded.

Randomisation

Participants will be randomised by opaque sealed envelope after enrolment. Numbers in the envelope will be generated by a computer-generated randomisation (http://www.randomization.com) table based on blocks of four to assign patients to intervention or usual care group. The randomization sequence will be concealed from consent designee staff and protected by an electronic password. Participants will be stratified based on a score > 3 on the Cumulative Illness rating Scale (CIRS-G) [29] We are stratifying patients as while most patients who develop sepsis syndromes have a number of co-morbidities, there can be a small number of relatively young, healthy patients who are affected. The allocation sequence will be generated by the research assistant attached to the trial. Patients will be consented by the chief investigator (JP) and assigned to interventions by a second investigator (PT).

INTERVENTION

Intervention Group

Patients in the intervention group will attend a follow-up clinic two-monthly for up to six months after discharge from the hospital. Screening instruments will be utilized on the first visit and appropriate management and referral provided. These will involve: overall medical review involving all systems assessment, medications, vital signs, physical activity assessment, muscle strength [30], assessment of mobility (*modified*

For peer review only - http://bmjopen.bmj.com/site/about/guidelines.xhtml

Rivermead Mobility Index,[31] balance assessment (*Berg Balance measure)* [32], discussion with carer as to concerns (*Zarit Burden interview),* [33] screening for chronic pain syndromes (*Brief Pain inventory*), [34] nutritional review , screening with the *(PTSD) Checklist-Civilian Version* (for post traumatic stress syndrome) [35] , *generalized anxiety disorder (GAD-7*) [36] and *The Patient Health Questionnaire-9 for depression* [37]. Following the results of the screening and team discussion, subjects and/or carer will be referred to appropriate agencies including general practitioner, specialist, physiotherapist, nutritionist, speech pathologist, exercise physiologist, psychologist and/or occupational therapist. These instruments are screening for post intensive care problems that have been identified in previous studies.[13-20] Members of the research team include an intensivist/thoracic physician, two specialist respiratory physiotherapists, a psychologist, a senior nurse researcher, a professor of community and general practice and a health economist/community physiotherapist. This individualized management is in line with recommendations from the NICE clinical guidelines [8].

Standard Care

Patients in the control group will have usual care ie referred to specialist outpatient clinics and/or general practitioners on discharge.

Sub Group - rural patients

A small number of patients (10) originally managed in the major quaternary level intensive care unit will be discharged to a rural area. We will test the feasibility of

For peer review only

providing follow-up care to these patients. The patients will also be randomized to either an intervention or usual care group. The patients will be interviewed via telemedicine (videoconferencing) [26] and a research assistant appointed to complete other screening tools and refer the patient to appropriate agencies. If specialist services are required eg chronic pain clinic, this may also be accessed via telemedicine.

OUTCOME MEASURES

Primary measure

The primary measure will be health related quality of life (HRQoL) as measured by the Short Form 36 health survey version 2 (SF36v2™)[38], which demonstrates content and construct validity, sensitivity and responsiveness to change in many patient groups[39] including intensive care survivors and post sepsis.[40] Pilot testing of the SF-36 in this population by this team has been conducted in a current trial of inpatients post sepsis and has shown excellent completion rates at six months by telephone.[41]

Secondary measures

Patients' readmission rates to hospital (medical record data), 12 month mortality (trend expected only) and economics and health care resource use. The latter will be monitored through examination of patient medical records, patient self-report and through Medicare / Pharmaceutical Benefits Scheme data extraction. The SF-6D[42] is a preference based six dimensional health state measure suitable for economic evaluation that is derived from the SF-36 The SF-6D defines 249 health states and a summary utility score for the SF-6D will be calculated using UK weights

For peer review only

Individual patient productivity will be measured using a work/activity impairment questionnaire, the Work Productivity and Activity Impairment Questionnaire (WPAI).[43]

The outcome measure of SF36v2™ will be taken at baseline, six and twelve months post enrolment in the study. All other measures (mortality, hospital readmission and economic and health resource use) will be taken at 6 and 12 months post enrolment.

Sample size

Power calculations are based on SF-36 using previously published Minimally Clinically Important Differences (MCIDs) for the SF36 physical domain scores of 7.8 points (sd 15) in our pilot trial on rehabilitation in sepsis.[41] The sample size, based on a two sample comparison of means was calculated at 78 per group for the SF-36 at 90% power with a two-sided, alpha error level of 0.05. Allowing for an almost 30% loss to follow up (mortality, drop outs) a total of 204 participants will be required.[44]

Planned Statistical analysis

Primary outcome

A baseline comparison of demographics, length of stay in intensive care, severity of illness acute physiological and chronic health evaluation score (APACHE II) on admission to intensive care, [45] and baseline measures will be done between each group, using a combination of t-tests and Chi-square.

Primary outcome: Generalized estimating equations (GEE) will be used to compare the SF-36 domain scores between groups and across time points. The GEE is a flexible technique that takes into account the correlations within individuals in

For peer review only - http://bmjopen.bmj.com/site/about/guidelines.xhtml

For peer review only

longitudinal study designs and can account for missing data without need for imputation techniques [46]

Secondary outcomes

Mortality will be compared between groups using a Cox proportional hazards (time until death) analysis proportional hazards (time until death) analysis.[47] Time to first readmission will be compared between groups by a survival analysis. Utility estimates derived from the SF-6D will be analysed using a GEE. Health care costs will be compared between groups using a generalized linear model (GLM) to account for the non normal distribution. Economic (health resource) evaluation will be a within trial incremental cost utility analysis with a societal perspective.[48] Direct costs included will be: cost of care provided in hospitals, by general practitioners, home nursing, complementary [allied] health providers, alternative health providers, opportunity cost of unpaid carers, cost of transportation to program and to other health services, and cost of pharmaceuticals. Indirect costs include loss of productivity due to disease, such as time lost from work or providing informal care to another individual. All costs will be valued using market costs where available and productivity costs will be valued using actual patient wage rates.

The sub group of rural patients will be analysed both in the full analysis and as a sub group results compared to metropolitan patients.

Data management

For peer review only

A custom designed data-base will store de-identified patient data on a secure password protected file. This will be entered from hard copies of the scoring sheets. Data will be reviewed by research office staff twice yearly and cross referenced with stored hard copies. A data management committee comprising a senior intensive care specialist, a senior critical care nurse and a senior physiotherapist will review the data every six months.

Contingencies

If subject recruitment does not reach the required sample size by two years, the study can be extended for a further one to two years.

Methods for protecting against other sources of bias

This study will be double blinded with the assessors, data analysts and a number of investigators (TC, FC, GM) blinded from the group allocation. There will be concealed allocation of subjects. The subjects will be stratified for Cumulative Illness rating Scale (CIRS-G). [29] Subjects when being consented will be told they will in one of two groups who will both be followed up post hospital and may need to attend an outpatient clinic. Patients are unaware of the usual follow up post hospital so should not feel they are receiving an increased/decreased level of care. Both groups will be contacted by telephone for completion of HRQoL life instruments at the required stages. Subjects may have varying severity of illness, lengths of stay and/or time on mechanical ventilation in intensive care, however this will be compared between groups and if unequal adjusted models using the relevant covariates will be presented as well as unadjusted comparisons.

For peer review only - http://bmjopen.bmj.com/site/about/guidelines.xhtml

A few subjects in either group may be referred to rehabilitation by their treating physician. This will be compared between groups. A number of patients may be uncontactable for final outcome measures however we have had 95 percent rate in a current trial at six months post sepsis. [41] The GEE [46] will allow for missing data. If patients “cross over” ie do not receive the planned intervention, they will still be analysed with the group they originally enrolled as per intention to treat.

ETHICS AND DISSEMINATION

The trial has been registered on the Australian and New Zealand Clinical Trials Registry (ACTRN12613000528752). Ethical approval has been granted from Royal Brisbane & Womens Hospital Human Research Ethics Committee (HREC) (HREC/13/QRBW/17), The University of Queensland HREC (2013000543), Griffith University (RHS/08/14/HREC) and the Australian Government Department of Health (26/2013).

Any adverse events connected to the trial would be immediately reported to one of the above three committees.

The results of this study will be submitted for publication to peer reviewed intensive care journals and presented at national and international intensive care and/or rehabilitation conferences.

CONCLUSION

For peer review only

Follow-up post intensive care stay is presently a topic of great interest with a realization that surviving critical illness may result in poor quality of life. Patients who have had severe sepsis syndromes have been shown to have a poorer quality of life and outcome than generic intensive care patients. This study will investigate whether targeted intervention from a multidisciplinary team will result in benefits and is economically feasible for this group of patients.

For peer review only

For peer review only - http://bmjopen.bmj.com/site/about/guidelines.xhtml

Funding: Funding has been provided for this study from the Royal Brisbane and Womens' Hospital Research Foundation Research foundation ($40,000) and the Physiotherapy Research Fellowship, Health and Medical Research, Queensland Government ($182,000).

Contributorship Statement: All authors participated in the manuscript preparation, writing and editing. We have added Dr Sunil Singh to the authors as he participated in grant writing and protocol development. It was an oversight that he was not on the original manuscript.

Competing interests: There are no competing interests

Data Sharing Statement: No additional data are available

For peer review only

References

1. Oeyen SG, Vandijck DM, Benoit DB et al. Quality of life after intensive care: a systematic review of the literature. *Crit Care Med* 2010;38:2386-400

2. Myhren H, Ekeberg O, Stokland O. Health related quality of life and return to work after critical illness in general intensive care unit patients: a 1-year follow up study. *Crit Care Med 2010*;38:1554-61

3. Williams TA, Dobb GJ, Finn JC et al. Long term survival from intensive care: a review. *Intensive Care Med* 2005;311306-15

4. Desai SV, Law TJ, Needham DM. Long-term complications of critical care. *Crit Care Med* 2011;39:371-9

5. Battle CE, Lovett S, Hutchings H. Chronic pain in survivors of critical illness: a retrospective analysis of incidence and risk factors. *Crit Care* 2013, 17:R101 http:// ccforum.com.contnet/17/3/R101

6. Griffiths J Hatch RA, Bishop J et al. An exploration of social and economic outcome and health related quality of life after critical illness in general intensive care unit survivors: a 12-month follow-up study. *Crit Care* 2013; 17:R100, http://ccforum.com/content/17/3/R100

7. Needham DM, Feldman DR, Kho ME. The functional costs of ICU survivorship. Collaborating to improve post-ICU disability. *Am J Respir Crit Care Med* 2011;183:1037-42

8. Rehabilitation after critical illness. NHS National Institute for Health and Clinical Excellence 2009, www.guidance.nice.org.uk/cg83

9. Hofhuis JGM, Spronk PE, van Stel HF et al. The impact of severe sepsis on health related quality of life: a long term follow up study. *Critical Care and Trauma* 2008;107:1957-64

10. Levy MM, Fink MP, Marshall JC et al. 2001 SCCM/ESICM/ACCP/ATS/SIS International Sepsis Definitions Conference. *Intensive Care Med* 2003;29:530–8

11. Angus DC, Lind-Zwirble WT, Lidicker J et al. Epidemiology of severe sepsis in the United States analysis of incidence outcome, and associated costs. *Crit Care Med* 2001;29:1303-10

12. Silva E, Araujo DV. Economic and Social Burden of Severe Sepsis. In Yearbook of Intensive Care and Emergency Medicine 2009:129-38

13. Karlsson S, Ruokonen E, Varpula T et al. Long-term outcome and quality adjusted life years after severe sepsis. *Crit Care Med* 2009; 37: 1268-74

14. Zhang K, Mao X, Fang Q, et al. Impaired long-term quality of life in survivors of severe sepsis Chinese multicenter study over 6 years. *Anaesthesist* 2013; 62:995–1002

15. Cuthbertson BH, Elder A, Hall S, et al. Mortality and quality of life in the five years after severe sepsis. Critical Care 2013, 17:R70 http:// ccforum.com/content/17/2/R70

16. Winters BD, Eberlein M, Leung J et al. Long-term mortality and quality of life in sepsis: a systematic review .*Crit Care Med* 2010;38:1276-83

17. Paratz JD, Kayambu G. Early exercise and attenuation of myopathy in the patient with sepsis in ICU. *Phys Ther Rev* 2011;16:58-65

For peer review only - http://bmjopen.bmj.com/site/about/guidelines.xhtml

18. Baldwin CE, Bersten AD. Alterations in respiratory and limb muscle strength and size in patients with sepsis who are mechanically ventilated. *Phys Ther* 2014;94:68-82.

19. Zimmer A, Rothaug J, Mescha S, et al. Chronic pain after surviving sepsis. *Crit Care* 2006: 10(suppl 1):421.

20. Iwashyna TJ, Ely EW, Smith DM et al. Long-term cognitive impairment and functional disability among survivors of severe sepsis. *JAMA* 2010;304: 1787-94

21. Griffiths JA, Barber VS, Cuthbertson BH et al. A national survey of intensive care follow-up clinics. *Anaesthesia* 2006;61:950–5

22. Bambi S, Solaro M, Cianchi G et al. Outpatient Intensive Care Unit Follow-Up Services in Italy: A National Survey A Brief Report. *Dimens Crit Care Nurs* 2013;32:46-9

23. Williams TA, Leslie GD. Beyond the walls: A review of ICU clinics and their impact on patient outcomes after leaving hospital. *Aust Critical Care* 2008;21:6-17

24. Cuthbertson BH, Rattray J, Campbell MK et al. The PRaCTICal study of nurse led, intensive care follow up programmes for improving long term outcomes from critical illness: a pragmatic randomised controlled trial. *BMJ* 2009; 339:b3723. doi: 10.1136/bmj.b3723.

25. Williams TA, Leslie GD. Challenges and possible solutions for long-term follow-up of patients surviving critical illness. *Aust Crit Care* 2011;24:175-85

26. Boots RJ, Singh S, Terblanche M et al. Remote care by telemedicine in the ICU: many models of care can be effective. *Curr Opin Crit Care*. 2011;17:634-40

27. Moher D, Schulz K, Altman D. The CONSORT statement: revised recommendations for improving the quality of parallel–group randomized controlled trials. *Ann Internal Med* 2001;134:657-62

28. Chan AW, Teztlaff JM, Gotzsche PC et al. SPIRIT 2013 explanation and elaboration: guidance for protocols of clinical trials. BMJ 2013;346:37586 doi:10.1136/bmj.e7586

29. Waldman E, Potter JF. A prospective evaluation of the Cumulative Illness Rating Scale. *Aging* 1992;4:171-8.

30. Baldwin CE, Paratz JD, Bersten AD. Muscle strength assessment in critically ill patients with handheld dynamometry: An investigation of reliability, minimal detectable change, and time to peak force generation. *J Crit Care* 2012;28:77-86

For peer review only

31. Lennon S, Johnson L. The modified Rivermead Mobility Index: validity and reliability. *Disabil Rehabil*. 2000;22:833–9.

32. Thorbahn LD, Newton RA. Use of the Berg Balance test to predict falls in elderly persons. *PTJ*. 1996;76:576–85.

33. Zarit SH, Reever KE, Bach-Peterson J. Relatives of the impaired elderly: correlates of feelings of burden. *Gerontologist* 1980;20:649-55.

34. Cleeland CS. Measurement of pain by subjective report. In: Chapman CR, Loeser JD, editors. *Advances in Pain Research and Therapy*, Volume 12: Issues in Pain Measurement. New York: Raven Press; 1989:391-403.

35. Conybeare D, Behar E, Solomon A et al. The PTSD Checklist—Civilian Version: Reliability, Validity, and Factor Structure in a Nonclinical Sample. *J Clin Psychol* 2012;68:699-713

36. Spitzer RL, Kroenke K, Williams JBW et al. A brief measure for assessing generalized anxiety disorder. *Arch Intern Med*. 2006;166:1092-7.

37. Kroenke K, Spitzer RL, Williams JBW. The PHQ-9 Validity of a Brief Depression Severity Measure. *J Gen Intern Med* 2001;16:606–13.

38. Ware JD, Sherbourne CD. The MOS 36-item short-form health survey (SF-36). I. Conceptual framework and item selection. *Medical Care* 1992;30:473-83.

39. Contopoulos-Ioannidis DG, Karvouni A, Kouri I et al. Reporting and interpretation of SF-36 outcomes in randomised trials: systematic review. *BMJ,* 2009; 338: doi: 10.1136/bmj.a3006.a3006.

40. Heyland DK, Hopman W, Coo H et al. Long-term health-related quality of life in survivors of sepsis. Short Form 36: A valid and reliable measure of health-related quality of life. *Crit Care Med* 2000;28:3599-605.

41. Kayambu G, Boots R, Paratz J. A Prospective Randomised Controlled Trial investigating Functional and Physiological Outcomes following Early Rehabilitation in Sepsis: The i-PERFORM Trial - Protocol Article. *BMC Anesthesiology* 2011;11:21. doi: 10.1186/1471-2253-11-21

42. Brazier J, Roberts J, Deverill M. The estimation of a preference-based measure of health from the SF-36. *J Health Economics* 2002;21:271-92.

43. Reilly MC, Zbrozek AS, Dukes E. The validity and reproducibility of a work productivity and activity impairment measure. *PharmacoEconomics* 1993; 4:353-65.

44. Cohen J. Statistical power analysis for the behavioral sciences. Hillsdale, NJ, Erlbaum 1988.

For peer review only

45. Knaus WA, Draper EA, Wagner DP et al. APACHE II: a severity of disease classification system. *Crit Care Med* 1985;13: 818–29

46. Twisk, J. de Vente W. Attrition in longitudinal studies. How to deal with missing data. *J Clin Epidemiol 2002;* 55:329-37

47. Schoenfeld D. Sample-size formula for the proportional-hazards regression model. *Biometrics* 1983;39:499-503.

48. Drummond M, O'Brien B, Stoddart G et al. methods for the economic evaluation of health care programmes. Oxford medical Publications. New York, 1993

For peer review only

For peer review only - http://bmjopen.bmj.com/site/about/guidelines.xhtml

IMPOSE: IMProving Outcomes after SEpsis: The effect of a multidisciplinary follow up service on health related quality of life in patients post sepsis syndromes, a double blinded randomized controlled trial: protocol

Investigators: Jennifer D Paratz[1,2,5,8], Justin Kenardy[3], Tracy Comans[4], Geoffrey Mitchell[6], Fiona Coyer[7], Peter Thomas[1,2,8], Sunil Singh[9], Louise Luparia[1,5], Robert J Boots [1,2]

[1]Burn, Trauma and Critical Care Research Centre, The University of Queensland; Brisbane, Australia [2]Department of Intensive Care Medicine, Royal Brisbane & Women's Hospital Brisbane, Australia; [3]CONROD, The University of Queensland Brisbane, Australia; [4]School of Medicine, Griffith University, Brisbane, Australia; [5]School of Rehabilitation Sciences, Griffith University, Brisbane, Australia [6]School of Medicine (Ipswich Campus), The University of Queensland, Ipswich, Australia; [7] Nursing Faculty, Queensland University of Technology Brisbane, Australia; [8]Department of Physiotherapy, Royal Brisbane & Women's Hospital; Brisbane, Australia, [9]Intensive Care Unit, Bundaberg Hospital, Bundaberg, Australia

Corresponding author:

Dr Jennifer Paratz

Chair and Senior Research Fellow
Burn, Trauma and Critical Care Research Centre
Department of Intensive Care Medicine
Level 3, Ned Hanlon Building
Royal Brisbane and Women's Hospital
Butterfield Street, Brisbane, QLD, 4029
Telephone: 61 7 3646 1980
Facsimile: 61 7 36463542
Email: j.paratz@uq.edu.au

Key words: sepsis, septic shock, critical care, intensive care, weakness, post traumatic stress syndrome

Word Count: 2,507

Trial registration number: ACTRN12613000528752

For peer review only

ABSTRACT

Introduction: Patients post sepsis syndromes have poor quality of life and a high rate of recurring illness or mortality. Follow-up clinics have been instituted for patients post general intensive care but evidence is sparse and there has been no clinic specifically for survivors of sepsis. The aim of this trial is to investigate if targeted screening and appropriate intervention to these subjects can result in an improved quality of life (Short Form -36 health survey (SF36v2), decreased mortality in the first 12 months, decreased readmission to hospital and/or decreased use of health resources.

Methods and Analysis: 204 patients post sepsis syndrome will be randomized to one of two groups. The intervention group will attend an outpatient clinic two-monthly for six months and receive screening and targeted intervention. The usual care group will remain under the care of their physician. To analyse the results a baseline comparison will be done between each group. Generalized estimating equations (GEE) will compare the SF-36 domain scores between groups and across time points. Mortality will be compared between groups using a Cox proportional hazards analysis proportional hazards (time until death) analysis. Time to first readmission will be compared between groups by a survival analysis. Health care costs will be compared between groups using a generalized linear model (GLM). Economic (health resource) evaluation will be a within trial incremental cost utility analysis with a societal perspective.

Ethics and Dissemination: Ethical approval has been granted from Royal Brisbane & Womens Hospital Human Research Ethics Committee (HREC) (HREC/13/QRBW/17), The University of Queensland HREC (2013000543), Griffith University (RHS/08/14/HREC) and the Australian Government Department of Health (26/2013). The results of this study will be submitted to peer reviewed intensive care journals and presented at national and international intensive care and/or rehabilitation conferences.

Australian and New Zealand Clinical Trials Registry ACTRN12613000528752

For peer review only

Article Summary

Article Focus

- Survivors of critical illness have been noted to have physical, psychological and social problems for up to five years post discharge.
- The subgroup of patients with sepsis syndromes have been noted to have worse outcome compared to general intensive care patients
- No studies have investigated targeted intervention for patients with sepsis syndromes post discharge from intensive care

Key Messages

- This study will investigate if providing screening and intervention to patients post sepsis syndrome will result in an improved quality of life, less readmission to hospital, less use of health resources and decreased mortality.

Strengths and limitations of this Study

- This is the first study to concentrate solely on patients with sepsis syndrome post intensive care discharge.
- There is a strong multidisciplinary team to provide a multitude of screening tools and interventions.
- Individualized management will be provided to patients.
- This is a single centre study so lacks external generalizibility

For peer review only

BACKGROUND

Greater numbers of patients are surviving intensive care (ICU) but a new syndrome of "post intensive care syndrome" has appeared with residual physical, cognitive and psychosocial problems lasting from 5 to 15 years.[1,2] The rate of recurring major illness and/or mortality is also increased in the year after ICU discharge, with mortality ranging from 26-63% at one year post discharge in long stay ICU patients (≥ 48 hours).[3] ICU survivors additionally often have a number of problems [1-4] including debility and fatigue from loss of muscle mass, ongoing nutritional problems, difficulty with dealing with altered appearance and function, chronic pain [5] amnesia, and delusional symptoms All of these factors have a major impact on the health and productivity of survivors and carers, return to work rates, as well as the impact on availability of ICU and hospital beds, surgical waiting lists, health costs and society.[6] There has been a "call to arms" to provide improved management regimes to intensive care survivors.[7] The NICE clinical guidelines [8] provided by the NHS National Institute of Health and Clinical Excellence recommend "optimisation of recovery" rather than merely survival post intensive care. All of these problems are particularly present in survivors of sepsis.[9]

Sepsis is defined as a powerful inflammatory response to severe infection. [10] The annual total costs of sepsis syndromes in the US are $16.7 billion nationally [11] with the incidence projected to increase by 1.5% per annum as patients develop more co-morbidities and resistant organisms. In brief, sepsis is a major public health concern.[12]

For peer review only

However the statistics above do not include the cost of ongoing disability and loss of productivity post illness. Patients with sepsis syndromes have significantly worse outcomes with lower health related quality of life (HRQoL)[13-16], particularly in the physical domain,[17,18] chronic pain [5,19] and cognitive domain [20] compared to non-septic ICU survivors. While other critically ill/injured or disabled patients eg burns, strokes, head injuries have established follow up regimes, patients with sepsis are not referred to formal rehabilitation and are discharged home with extremely poor function.

Follow up intensive care outpatient clinics are a recent innovation particularly within the United Kingdom. [21-23] These clinics identify and provide intervention for problems post hospital discharge including weakness, poor balance, impaired swallowing and nutrition, ongoing emotional problems and/or post-traumatic stress disorder in critical illness survivors. The one completed clinical trial on this topic [24] did not find a significant improvement in HRQoL. Researchers admitted that one discipline was not ideal and "further work should focus on the roles of early physical rehabilitation, delirium, cognitive dysfunction and relatives". The programme was conducted in a generic group of ICU survivors including those with only an overnight stay, and there may be greater discriminatory power in selecting one diagnostic group with a documented poor outcome such as sepsis syndromes. Additionally some countries (Canada, Australia, South Africa) have major problems with large land area and relatively sparse population in rural areas leading to lack of resources and problems with follow up post intensive care. [25]

AIMS

For peer review only

The primary aim of this research is to investigate whether targeted follow up and intervention will improve health related quality of life (HRQoL) in survivors of sepsis syndromes. Secondary aims will be to investigate whether this intervention decreases readmission rates to hospital, 12 month mortality and health resource use.

This project will be an important investigation of follow up and management of the ICU patient in a sub population (sepsis) documented to have poor outcomes.[16] The project will incorporate a subgroup (rural patients) via telemedicine [26] in a novel method to overcome the problem of the "tyranny of distance" in follow up.

Hypotheses

The hypotheses are that post hospital follow up will improve health related quality of life, decrease mortality, economic and health resource use and readmission to hospital in patients post admission to intensive care with sepsis syndrome.

METHODS/DESIGN

This is a prospective, double blinded, interventional, repeated measures, superiority, randomised controlled trial with concealed allocation, blinded assessors and intention to treat analysis. The study has been designed with the Consolidated Standards for Reporting of Trials guidelines [27] (Figure 1) and the Standard Protocol items: Recommendation for Interventional Trials (SPIRIT 2013). [28] The trial will be completed in two outpatient clinics. One clinic will be in the outpatient department located at a university-affiliated tertiary hospital Royal Brisbane & Womens Hospital,

For peer review only

Brisbane, Australia; the other will be located at a regional hospital, the Bundaberg Base Hospital, Bundaberg, Queensland, Australia

Subjects

Participants will be recruited from patients being discharged from a quaternary university-affiliated intensive-care unit at Royal Brisbane and Women's Hospital (RBWH), Brisbane, Australia. Participants will be randomized to one of two groups (intervention or control) post ICU discharge and just prior to discharge from hospital.

Inclusion criteria

Patients included will be male or female, ≥18 years, with a documented episode(s) of sepsis [10] episode(s) of sepsis (≥2 criteria of a systemic inflammatory response plus proven or strongly suspected infection, severe sepsis defined as sepsis plus organ failure, septic shock defined as severe sepsis not responding to management) and required respiratory support for longer than 48 hours. Enrolment in the study should be within one month of discharge from hospital.

Exclusion criteria

Patients with neurological injuries, spinal injuries, and burns will be excluded, as these patients have existing rehabilitation programs and community support groups. Patients with haematological conditions or requiring palliative care post ICU would also be excluded. Those or mental disabilities that patients with psychiatric

For peer review only

and/mental disability that preclude them understanding the questionnaires and non-English speaking patients would be excluded.

Randomisation

Participants will be randomised by opaque sealed envelope after enrolment. Numbers in the envelope will be generated by a computer-generated randomisation (http://www.randomization.com) table based on blocks of four to assign patients to intervention or usual care group. The randomization sequence will be concealed from consent designee staff and protected by an electronic password. Participants will be stratified based on a score > 3 on the Cumulative Illness rating Scale (CIRS-G) [29] We are stratifying patients as while most patients who develop sepsis syndromes have a number of co-morbidities, there can be a small number of relatively young, healthy patients who are affected. The allocation sequence will be generated by the research assistant attached to the trial. Patients will be consented by the chief investigator (JP) and assigned to interventions by a second investigator (PT).

INTERVENTION

Intervention Group

Patients in the intervention group will attend a follow-up clinic two-monthly for up to six months after discharge from the hospital. Screening instruments will be utilized on the first visit and appropriate management and referral provided. These will involve: overall medical review involving all systems assessment, medications, vital signs, physical activity assessment, muscle strength [30], assessment of mobility (*modified*

For peer review only

Rivermead Mobility Index,[31] balance assessment (*Berg Balance measure)* [32], discussion with carer as to concerns (*Zarit Burden interview),* [33] screening for chronic pain syndromes (*Brief Pain inventory*), [34] nutritional review , screening with the *(PTSD) Checklist-Civilian Version* (for post traumatic stress syndrome) [35] , *generalized anxiety disorder (GAD-7*) [36] and *The Patient Health Questionnaire-9 for depression* [37]. Following the results of the screening and team discussion, subjects and/or carer will be referred to appropriate agencies including general practitioner, specialist, physiotherapist, nutritionist, speech pathologist, exercise physiologist, psychologist and/or occupational therapist. These instruments are screening for post intensive care problems that have been identified in previous studies.[13-20] Members of the research team include an intensivist/thoracic physician, two specialist respiratory physiotherapists, a psychologist, a senior nurse researcher, a professor of community and general practice and a health economist/community physiotherapist. This individualized management is in line with recommendations from the NICE clinical guidelines [8].

Standard Care

Patients in the control group will have usual care ie referred to specialist outpatient clinics and/or general practitioners on discharge.

Sub Group - rural patients

A small number of patients (10) originally managed in the major quaternary level intensive care unit will be discharged to a rural area. We will test the feasibility of

For peer review only

providing follow-up care to these patients. The patients will also be randomized to either an intervention or usual care group. The patients will be interviewed via telemedicine (videoconferencing) [26] and a research assistant appointed to complete other screening tools and refer the patient to appropriate agencies. If specialist services are required eg chronic pain clinic, this may also be accessed via telemedicine.

OUTCOME MEASURES

Primary measure

The primary measure will be health related quality of life (HRQoL) as measured by the Short Form 36 health survey version 2 (SF36v2™)[38], which demonstrates content and construct validity, sensitivity and responsiveness to change in many patient groups[39] including intensive care survivors and post sepsis.[40] Pilot testing of the SF-36 in this population by this team has been conducted in a current trial of inpatients post sepsis and has shown excellent completion rates at six months by telephone.[41]

Secondary measures

Patients' readmission rates to hospital (medical record data), 12 month mortality (trend expected only) and economics and health care resource use. The latter will be monitored through examination of patient medical records, patient self-report and through Medicare / Pharmaceutical Benefits Scheme data extraction. The SF-6D[42] is a preference based six dimensional health state measure suitable for economic evaluation that is derived from the SF-36 The SF-6D defines 249 health states and a summary utility score for the SF-6D will be calculated using UK weights

For peer review only - http://bmjopen.bmj.com/site/about/guidelines.xhtml

Individual patient productivity will be measured using a work/activity impairment questionnaire, the Work Productivity and Activity Impairment Questionnaire (WPAI).[43]

The outcome measure of SF36v2™ will be taken at baseline, six and twelve months post enrolment in the study. All other measures (mortality, hospital readmission and economic and health resource use) will be taken at 6 and 12 months post enrolment.

Sample size

Power calculations are based on SF-36 using previously published Minimally Clinically Important Differences (MCIDs) for the SF36 physical domain scores of 7.8 points (sd 15) in our pilot trial on rehabilitation in sepsis.[41] The sample size, based on a two sample comparison of means was calculated at 78 per group for the SF-36 at 90% power with a two-sided, alpha error level of 0.05. Allowing for an almost 30% loss to follow up (mortality, drop outs) a total of 204 participants will be required.[44]

Planned Statistical analysis

Primary outcome

A baseline comparison of demographics, length of stay in intensive care, severity of illness acute physiological and chronic health evaluation score (APACHE II) on admission to intensive care, [45] and baseline measures will be done between each group, using a combination of t-tests and Chi-square.

Primary outcome: Generalized estimating equations (GEE) will be used to compare the SF-36 domain scores between groups and across time points. The GEE is a flexible technique that takes into account the correlations within individuals in

For peer review only - http://bmjopen.bmj.com/site/about/guidelines.xhtml

longitudinal study designs and can account for missing data without need for imputation techniques [46]

Secondary outcomes

Mortality will be compared between groups using a Cox proportional hazards (time until death) analysis proportional hazards (time until death) analysis.[47] Time to first readmission will be compared between groups by a survival analysis. Utility estimates derived from the SF-6D will be analysed using a GEE. Health care costs will be compared between groups using a generalized linear model (GLM) to account for the non normal distribution. Economic (health resource) evaluation will be a within trial incremental cost utility analysis with a societal perspective.[48] Direct costs included will be: cost of care provided in hospitals, by general practitioners, home nursing, complementary [allied] health providers, alternative health providers, opportunity cost of unpaid carers, cost of transportation to program and to other health services, and cost of pharmaceuticals. Indirect costs include loss of productivity due to disease, such as time lost from work or providing informal care to another individual. All costs will be valued using market costs where available and productivity costs will be valued using actual patient wage rates.

The sub group of rural patients will be analysed both in the full analysis and as a sub group results compared to metropolitan patients.

Data management

For peer review only

A custom designed data-base will store de-identified patient data on a secure password protected file. This will be entered from hard copies of the scoring sheets. Data will be reviewed by research office staff twice yearly and cross referenced with stored hard copies. A data management committee comprising a senior intensive care specialist, a senior critical care nurse and a senior physiotherapist will review the data every six months.

Contingencies

If subject recruitment does not reach the required sample size by two years, the study can be extended for a further one to two years.

Methods for protecting against other sources of bias

This study will be double blinded with the assessors, data analysts and a number of investigators (TC, FC, GM) blinded from the group allocation. There will be concealed allocation of subjects. The subjects will be stratified for Cumulative Illness rating Scale (CIRS-G). [29] Subjects when being consented will be told they will in one of two groups who will both be followed up post hospital and may need to attend an outpatient clinic. Patients are unaware of the usual follow up post hospital so should not feel they are receiving an increased/decreased level of care. Both groups will be contacted by telephone for completion of HRQoL life instruments at the required stages. Subjects may have varying severity of illness, lengths of stay and/or time on mechanical ventilation in intensive care, however this will be compared between groups and if unequal adjusted models using the relevant covariates will be presented as well as unadjusted comparisons.

For peer review only

A few subjects in either group may be referred to rehabilitation by their treating physician. This will be compared between groups. A number of patients may be uncontactable for final outcome measures however we have had 95 percent rate in a current trial at six months post sepsis. [41] The GEE [46] will allow for missing data. If patients "cross over" ie do not receive the planned intervention, they will still be analysed with the group they originally enrolled as per intention to treat.

ETHICS AND DISSEMINATION

The trial has been registered on the Australian and New Zealand Clinical Trials Registry (ACTRN12613000528752). Ethical approval has been granted from Royal Brisbane & Womens Hospital Human Research Ethics Committee (HREC) (HREC/13/QRBW/17), The University of Queensland HREC (2013000543), Griffith University (RHS/08/14/HREC) and the Australian Government Department of Health (26/2013).

Any adverse events connected to the trial would be immediately reported to one of the above three committees.

The results of this study will be submitted for publication to peer reviewed intensive care journals and presented at national and international intensive care and/or rehabilitation conferences.

CONCLUSION

For peer review only

Follow-up post intensive care stay is presently a topic of great interest with a realization that surviving critical illness may result in poor quality of life. Patients who have had severe sepsis syndromes have been shown to have a poorer quality of life and outcome than generic intensive care patients. This study will investigate whether targeted intervention from a multidisciplinary team will result in benefits and is economically feasible for this group of patients.

Competing interests: Nil

Funding: Funding has been provided for this study from the Royal Brisbane and Womens' Hospital Research Foundation Research foundation ($40,000) and the Physiotherapy Research Fellowship, Health and Medical Research, Queensland Government ($182,000).

For peer review only

References

1. Oeyen SG, Vandijck DM, Benoit DB et al. Quality of life after intensive care: a systematic review of the literature. *Crit Care Med* 2010;38:2386-400

2. Myhren H, Ekeberg O, Stokland O. Health related quality of life and return to work after critical illness in general intensive care unit patients: a 1-year follow up study. *Crit Care Med 2010*;38:1554-61

3. Williams TA, Dobb GJ, Finn JC et al. Long term survival from intensive care: a review. *Intensive Care Med* 2005;311306-15

4. Desai SV, Law TJ, Needham DM. Long-term complications of critical care. *Crit Care Med* 2011;39:371-9

5. Battle CE, Lovett S, Hutchings H. Chronic pain in survivors of critical illness: a retrospective analysis of incidence and risk factors. *Crit Care* 2013, 17:R101 http:// ccforum.com.contnet/17/3/R101

6. Griffiths J Hatch RA, Bishop J et al. An exploration of social and economic outcome and health related quality of life after critical illness in general intensive care unit survivors: a 12-month follow-up study. *Crit Care* 2013; 17:R100, http://ccforum.com/content/17/3/R100

7. Needham DM, Feldman DR, Kho ME. The functional costs of ICU survivorship. Collaborating to improve post-ICU disability. *Am J Respir Crit Care Med* 2011;183:1037-42

8. Rehabilitation after critical illness. NHS National Institute for Health and Clinical Excellence 2009, www.guidance.nice.org.uk/cg83

9. Hofhuis JGM, Spronk PE, van Stel HF et al. The impact of severe sepsis on health related quality of life: a long term follow up study. *Critical Care and Trauma* 2008;107:1957-64

10. Levy MM, Fink MP, Marshall JC et al. 2001 SCCM/ESICM/ACCP/ATS/SIS International Sepsis Definitions Conference. *Intensive Care Med* 2003;29:530–8

11. Angus DC, Lind-Zwirble WT, Lidicker J et al. Epidemiology of severe sepsis in the United States analysis of incidence outcome, and associated costs. *Crit Care Med* 2001;29:1303-10

12. Silva E, Araujo DV. Economic and Social Burden of Severe Sepsis. In Yearbook of Intensive Care and Emergency Medicine 2009:129-38

13. Karlsson S, Ruokonen E, Varpula T et al. Long-term outcome and quality adjusted life years after severe sepsis. *Crit Care Med* 2009; 37: 1268-74

14. Zhang K, Mao X, Fang Q, et al. Impaired long-term quality of life in survivors of severe sepsis Chinese multicenter study over 6 years. *Anaesthesist* 2013; 62:995–1002

For peer review only

15. Cuthbertson BH, Elder A, Hall S, et al. Mortality and quality of life in the five years after severe sepsis. Critical Care 2013, 17:R70 http://ccforum.com/content/17/2/R70

16. Winters BD, Eberlein M, Leung J et al. Long-term mortality and quality of life in sepsis: a systematic review .*Crit Care Med* 2010;38:1276-83

17. Paratz JD, Kayambu G. Early exercise and attenuation of myopathy in the patient with sepsis in ICU. *Phys Ther Rev* 2011;16:58-65

18. Baldwin CE, Bersten AD. Alterations in respiratory and limb muscle strength and size in patients with sepsis who are mechanically ventilated. *Phys Ther* 2014;94:68-82.

19. Zimmer A, Rothaug J, Mescha S, et al. Chronic pain after surviving sepsis. *Crit Care* 2006: 10(suppl 1):421.

20. Iwashyna TJ, Ely EW, Smith DM et al. Long-term cognitive impairment and functional disability among survivors of severe sepsis. *JAMA* 2010;304: 1787-94

21. Griffiths JA, Barber VS, Cuthbertson BH et al. A national survey of intensive care follow-up clinics. *Anaesthesia* 2006;61:950–5

22. Bambi S, Solaro M, Cianchi G et al. Outpatient Intensive Care Unit Follow-Up Services in Italy: A National Survey A Brief Report. *Dimens Crit Care Nurs* 2013;32:46-9

23. Williams TA, Leslie GD. Beyond the walls: A review of ICU clinics and their impact on patient outcomes after leaving hospital. *Aust Critical Care* 2008;21:6-17

24. Cuthbertson BH, Rattray J, Campbell MK et al. The PRaCTICal study of nurse led, intensive care follow up programmes for improving long term outcomes from critical illness: a pragmatic randomised controlled trial. *BMJ* 2009; 339:b3723. doi: 10.1136/bmj.b3723.

25. Williams TA, Leslie GD. Challenges and possible solutions for long-term follow-up of patients surviving critical illness. *Aust Crit Care* 2011;24:175-85

26. Boots RJ, Singh S, Terblanche M et al. Remote care by telemedicine in the ICU: many models of care can be effective. *Curr Opin Crit Care*. 2011;17:634-40

27. Moher D, Schulz K, Altman D. The CONSORT statement: revised recommendations for improving the quality of parallel–group randomized controlled trials. *Ann Internal Med* 2001;134:657-62

For peer review only - http://bmjopen.bmj.com/site/about/guidelines.xhtml

28. Chan AW, Teztlaff JM, Gotzsche PC et al. SPIRIT 2013 explanation and elaboration: guidance for protocols of clinical trials. BMJ 2013;346:37586 doi:10.1136/bmj.e7586

29. Waldman E, Potter JF. A prospective evaluation of the Cumulative Illness Rating Scale. *Aging* 1992;4:171-8.

30. Baldwin CE, Paratz JD, Bersten AD. Muscle strength assessment in critically ill patients with handheld dynamometry: An investigation of reliability, minimal detectable change, and time to peak force generation. *J Crit Care* 2012;28:77-86

31. Lennon S, Johnson L. The modified Rivermead Mobility Index: validity and reliability. *Disabil Rehabil*. 2000;22:833–9.

32. Thorbahn LD, Newton RA. Use of the Berg Balance test to predict falls in elderly persons. *PTJ*. 1996;76:576–85.

33. Zarit SH, Reever KE, Bach-Peterson J. Relatives of the impaired elderly: correlates of feelings of burden. *Gerontologist* 1980;20:649-55.

34. Cleeland CS. Measurement of pain by subjective report. In: Chapman CR, Loeser JD, editors. *Advances in Pain Research and Therapy*, Volume 12: Issues in Pain Measurement. New York: Raven Press; 1989:391-403.

35. Conybeare D, Behar E, Solomon A et al. The PTSD Checklist—Civilian Version: Reliability, Validity, and Factor Structure in a Nonclinical Sample. *J Clin Psychol* 2012;68:699-713

36. Spitzer RL, Kroenke K, Williams JBW et al. A brief measure for assessing generalized anxiety disorder. *Arch Intern Med*. 2006;166:1092-7.

37. Kroenke K, Spitzer RL, Williams JBW. The PHQ-9 Validity of a Brief Depression Severity Measure. *J Gen Intern Med* 2001;16:606–13.

38. Ware JD, Sherbourne CD. The MOS 36-item short-form health survey (SF-36). I. Conceptual framework and item selection. *Medical Care* 1992;30:473-83.

39. Contopoulos-Ioannidis DG, Karvouni A, Kouri I et al. Reporting and interpretation of SF-36 outcomes in randomised trials: systematic review. *BMJ,* 2009; 338: doi: 10.1136/bmj.a3006.a3006.

40. Heyland DK, Hopman W, Coo H et al. Long-term health-related quality of life in survivors of sepsis. Short Form 36: A valid and reliable measure of health-related quality of life. *Crit Care Med* 2000;28:3599-605.

41. Kayambu G, Boots R, Paratz J. A Prospective Randomised Controlled Trial investigating Functional and Physiological Outcomes following Early

For peer review only

Rehabilitation in Sepsis: The i-PERFORM Trial - Protocol Article. *BMC Anesthesiology* 2011;11:21. doi: 10.1186/1471-2253-11-21

42. Brazier J, Roberts J, Deverill M. The estimation of a preference-based measure of health from the SF-36. *J Health Economics* 2002;21:271-92.

43. Reilly MC, Zbrozek AS, Dukes E. The validity and reproducibility of a work productivity and activity impairment measure. *PharmacoEconomics* 1993; 4:353-65.

44. Cohen J. Statistical power analysis for the behavioral sciences. Hillsdale, NJ, Erlbaum 1988.

45. Knaus WA, Draper EA, Wagner DP et al. APACHE II: a severity of disease classification system. *Crit Care Med* 1985;13: 818–29

46. Twisk, J. de Vente W. Attrition in longitudinal studies. How to deal with missing data. *J Clin Epidemiol 2002;* 55:329-37

47. Schoenfeld D. Sample-size formula for the proportional-hazards regression model. *Biometrics* 1983;39:499-503.

48. Drummond M, O'Brien B, Stoddart G et al. methods for the economic evaluation of health care programmes. Oxford medical Publications. New York, 1993

For peer review only

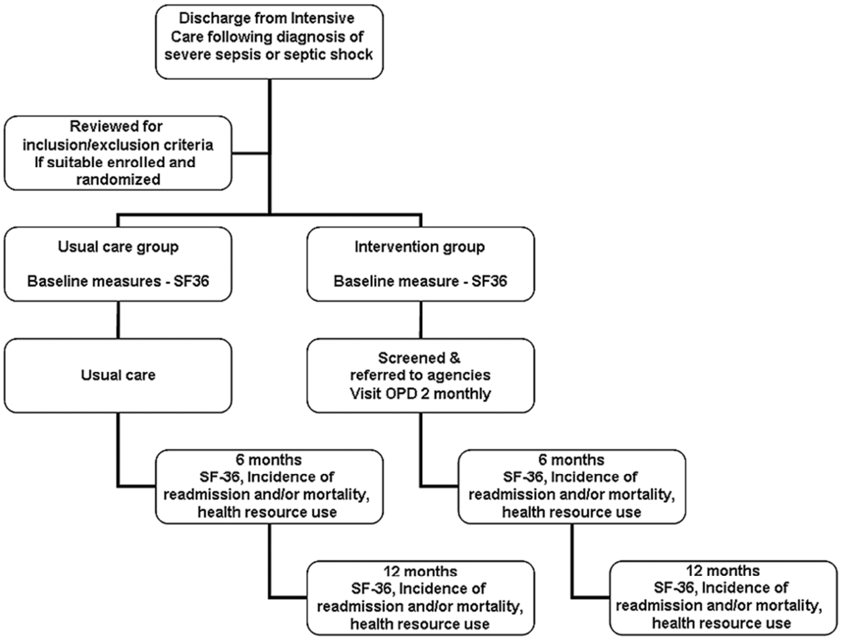

90x64mm (300 x 300 DPI)

view only

CONSORT 2010 checklist of information to include when reporting a randomised trial*

Section/Topic	Item No	Checklist item	Reported on page No
**Title and abstract**			
	1a	Identification as a randomised trial in the title	1
	1b	Structured summary of trial design, methods, results, and conclusions (for specific guidance see CONSORT for abstracts)	2,3
**Introduction**			
Background and objectives	2a	Scientific background and explanation of rationale	5,6
	2b	Specific objectives or hypotheses	7
**Methods**			
Trial design	3a	Description of trial design (such as parallel, factorial) including allocation ratio	7,8
	3b	Important changes to methods after trial commencement (such as eligibility criteria), with reasons	N/A
Participants	4a	Eligibility criteria for participants	8,9
	4b	Settings and locations where the data were collected	11
Interventions	5	The interventions for each group with sufficient details to allow replication, including how and when they were actually administered	9,10
Outcomes	6a	Completely defined pre-specified primary and secondary outcome measures, including how and when they were assessed	11,12
	6b	Any changes to trial outcomes after the trial commenced, with reasons	N/A
Sample size	7a	How sample size was determined	12
	7b	When applicable, explanation of any interim analyses and stopping guidelines	N/A
Randomisation:			
Sequence generation	8a	Method used to generate the random allocation sequence	9
	8b	Type of randomisation; details of any restriction (such as blocking and block size)	9
Allocation concealment mechanism	9	Mechanism used to implement the random allocation sequence (such as sequentially numbered containers), describing any steps taken to conceal the sequence until interventions were assigned	9
Implementation	10	Who generated the random allocation sequence, who enrolled participants, and who assigned participants to interventions	9
Blinding	11a	If done, who was blinded after assignment to interventions (for example, participants, care providers, those	14,15

For peer review only - http://bmjopen.bmj.com/site/about/guidelines.xhtml

		assessing outcomes) and how	
	11b	If relevant, description of the similarity of interventions	N/A
Statistical methods	12a	Statistical methods used to compare groups for primary and secondary outcomes	12,13
	12b	Methods for additional analyses, such as subgroup analyses and adjusted analyses	N/A
**Results**			
Participant flow (a diagram is strongly recommended)	13a	For each group, the numbers of participants who were randomly assigned, received intended treatment, and were analysed for the primary outcome	N/A
	13b	For each group, losses and exclusions after randomisation, together with reasons	N/A
Recruitment	14a	Dates defining the periods of recruitment and follow-up	N/A
	14b	Why the trial ended or was stopped	N/A
Baseline data	15	A table showing baseline demographic and clinical characteristics for each group	N/A
Numbers analysed	16	For each group, number of participants (denominator) included in each analysis and whether the analysis was by original assigned groups	N/A
Outcomes and estimation	17a	For each primary and secondary outcome, results for each group, and the estimated effect size and its precision (such as 95% confidence interval)	N/A
	17b	For binary outcomes, presentation of both absolute and relative effect sizes is recommended	N/A
Ancillary analyses	18	Results of any other analyses performed, including subgroup analyses and adjusted analyses, distinguishing pre-specified from exploratory	N/A
Harms	19	All important harms or unintended effects in each group (for specific guidance see CONSORT for harms)	N/A
**Discussion**			
Limitations	20	Trial limitations, addressing sources of potential bias, imprecision, and, if relevant, multiplicity of analyses	14,15
Generalisability	21	Generalisability (external validity, applicability) of the trial findings	N/A
Interpretation	22	Interpretation consistent with results, balancing benefits and harms, and considering other relevant evidence	N/A
**Other information**			
Registration	23	Registration number and name of trial registry	1,3
Protocol	24	Where the full trial protocol can be accessed, if available	Author
Funding	25	Sources of funding and other support (such as supply of drugs), role of funders	16

*We strongly recommend reading this statement in conjunction with the CONSORT 2010 Explanation and Elaboration for important clarifications on all the items. If relevant, we also recommend reading CONSORT extensions for cluster randomised trials, non-inferiority and equivalence trials, non-pharmacological treatments, herbal interventions, and pragmatic trials. Additional extensions are forthcoming: for those and for up to date references relevant to this checklist, see www.consort-statement.org.

For peer review only

For peer review only - http://bmjopen.bmj.com/site/about/guidelines.xhtml
